# Perioperative fasting: A time to relook

**DOI:** 10.4103/0019-5049.71021

**Published:** 2010

**Authors:** M Subrahmanyam, M Venugopal

**Affiliations:** Global Hospitals, Hyderabad, India

The article “Nil per oral after midnight: Is it necessary for clear fluids?” in this issue by Kajal Dalal draws our attention towards our age-old practice of fasting patients in the perioperative period. In the last decade, there has been mounting evidence that a rigid approach towards pre- and post-operative fasting may not be as beneficial as it was perceived to be. This article compels us to actually take a fresh look at our policies regarding fasting and anaesthesia.

It has been a routine practice to starve patients for 6–8 h prior to anaesthesia to reduce the risk of aspiration. But, there has been increasing evidence doubting the benefit such a practice imparts towards preventing aspiration. Both randomized control trials and reviews in the past decade have shown that there is little or no evidence that the volume or pH of gastric contents differed significantly whether patients were permitted a shortened pre-operative fluid fast or had a standard fast.[1] It has been observed that a rigid approach to fasting not only results in discomfort to the patients but also leads to blanket orders such as nil-per-oral after midnight, leading, in some instances, to fasting durations of 10–15 h. The American Society of Anesthesiology took the first step towards liberalizing pre-operative fasting guidelines by advocating clear liquids (non-particulate) orally 2 h prior to anaesthesia.[2] Application of these liberalized guidelines for pre-operative fasting and fluid intake has not resulted in increased pulmonary aspiration, morbidity or mortality. On the contrary, it has decreased irritability, anxiety, thirst and hunger in the perioperative period. Shortening of pre-operative fasting with the supply of a carbohydrate-rich beverage (non-particulate) up to 2 h before the operation has been shown to reduce insulin resistance and surgical stress and, additionally, improve the patient’s well-being.[3] Thus, having a more liberal starvation policy towards all patients who are not at a high risk of aspiration might actually be beneficial. But, there has been a lack of implementation of these liberalized guidelines into actual practice.

Apart from pre-operative fasting, it is time we also question the age-old practice of starving all patients post-operatively for extended periods of time after the anaesthetic has worn-off. There is no specific evidence or guideline that suggests a blanket practice of post-operative fasting. This kind of fasting was justified in the days of ether and nitrous oxide anaesthesia that used to have profound post-operative nausea and vomiting (PONV). There is no justification to deny anyone a feed post-operatively unless there is actual bowel handling. Using air instead of nitrous oxide and minimizing the use of opioids by the simultaneous use of intravenous paracetamol and non-steroidal anti-inflammatory drugs help in reducing the incidence of PONV and post-operative gastrointestinal dysfunction. The use of ultrasound and nerve stimulators has made regional anaesthesia more effective and easier to administer. This has lead to an increasing utilization of regional techniques and reduction in the use of opioids and other drugs that affect gastrointestinal function. Thus, with regional techniques, we should now be able to feed patients much earlier than ever before. In fact, the authors routinely feed caesarean section patients within 1 h of surgery.

Interestingly, in the last decade, large reviews have highlighted the possible benefits of early post-operative feeding in patients after colorectal surgery,[4] major gynaecological surgery[5] and caesarean sections.[6] Early post-operative feeding in children and day-care patients is now routine practice. In this era of fast-tracking, where we have a tremendous pressure on resource utilization, anything that reduces the morbidity of patients and the length of hospital stay is immensely beneficial. Early feeding has been suggested to be one of the important components of a multi-modal approach towards fast-tracking patients.[7] Practices like reduced manipulation of the bowel, epidural anaesthesia and early enteral feeding itself can reduce the incidence of post-operative gastrointestinal dysfunction, in turn reducing morbidity and length of hospital stay.[8,9] This can significantly decrease health care costs while increasing patient satisfaction.

Thus, the emerging evidence suggests that we may be overstarving patients in the perioperative period. All patients except those who are at a high risk for aspiration should have a more liberal pre-operative fasting protocol. All patients (other than where the bowel was handled) should be allowed to feed post-operatively as early as possible after ensuring return of airway reflexes. To improve these perioperative practices related to fasting and feeding, anaesthesiologists, surgeons and the nursing staff must work together. Increasing awareness needs to be created among them. Further research regarding various aspects of perioperative fasting should be encouraged so that a lot of unanswered questions can be resolved.
